# Chromosome-Contiguous *Ancylostoma duodenale* Reference Genome from a Single Archived Specimen Elucidates Human Hookworm Biology and Host–Parasite Interactions †

**DOI:** 10.3390/ijms26125576

**Published:** 2025-06-11

**Authors:** Neil D. Young, Yuanting Zheng, Sunita B. Sumanam, Tao Wang, Jiangning Song, Bill C. H. Chang, Robin B. Gasser

**Affiliations:** 1 Department of Veterinary Biosciences, Melbourne Veterinary School, The University of Melbourne, Parkville, VIC 3010, Australia; yuanting.zheng@student.unimelb.edu.au (Y.Z.); sunita.sumanam@unimelb.edu.au (S.B.S.); tao.wang1@unimelb.edu.au (T.W.); bchang@ozomics.com (B.C.H.C.); 2 Biomedicine Discovery Institute and Department of Biochemistry and Molecular Biology, Monash University, Clayton, VIC 3800, Australia; jiangning.song@monash.edu; 3 Monash Data Futures Institute, Monash University, Clayton, VIC 3800, Australia

**Keywords:** hookworm disease, *Ancylostoma duodenale*, archived samples, third-generation sequencing, complete reference genome from a single worm, biology, host-parasite interactions

## Abstract

Soil-transmitted helminths (STHs) are parasitic nematodes that infect humans, particularly in tropical and subtropical regions, where they contribute substantially to neglected tropical diseases (NTDs). Among them, hookworms (*Ancylostoma duodenale*, *Necator americanus* and *Ancylostoma ceylanicum*) cause substantial morbidity, leading to anaemia, malnutrition, and developmental impairment. Despite the global impact of hookworm disease, genomic research on *A. duodenale* has lagged behind that of other hookworms, limiting comparative and molecular biological investigations. Here, we report the first chromosome-level reference genome of *A. duodenale*, assembled from a single adult specimen archived in ethanol at −20 °C for more than 27 years. Using third-generation sequencing (PacBio Revio, Menlo Park, CA, USA, Oxford Nanopore, Oxford, UK), Hi-C scaffolding, and advanced computational tools, we produced a high-quality 319 Mb genome, filling a critical gap in hookworm genomics. Comparative analyses with *N. americanus* and the related, free-living nematode *Caenorhabditis elegans* provided new insights into genome organisation, synteny, and specific adaptations. While *A. duodenale* exhibited strong chromosomal synteny with *N. americanus*, its limited synteny with *C. elegans* highlights its distinct parasitic adaptations. We identified 20,015 protein-coding genes, including conserved single-copy orthologues (SCOs) linked to host–pathogen interactions, immune evasion and essential biological processes. The first comprehensive secretome analysis of *A. duodenale* revealed a diverse repertoire of excretory/secretory (ES) proteins, including immunomodulatory candidates predicted to interact with host structural and immune-related proteins. This study advances hookworm genomics, establishes a basis for the sequencing of archival specimens, and provides fundamental insights into the molecular biology of *A. duodenale*. The genomic resource for this hookworm species creates new opportunities for diagnostic, therapeutic, and vaccine development within a *One Health* framework. It complements recent epidemiological work and aligns with the WHO NTD roadmap (2021–2030) and Sustainable Development Goal 3.3.

## 1. Introduction

Soil-transmitted helminths (STHs) include a group of parasitic nematodes that infect humans, primarily in subtropical and tropical regions, particularly in low- and middle-income countries [1]. These parasites are responsible for some of the most significant neglected tropical diseases (NTDs) in terms of morbidity, with approximately one billion people infected with at least one species. STHs affecting humans include the common roundworm (*Ascaris* spp.), whipworm (*Trichuris* spp.), and blood-feeding hookworms (*Necator* and *Ancylostoma* spp.) [2].

Disease caused by hookworms remains a major public health concern, particularly in resource-limited regions where STHs contribute substantially to morbidity [3,4]. Of the predominant hookworm species that infect humans, *Necator americanus*, *Ancylostoma duodenale*, and *Ancylostoma ceylanicum* are responsible for a major disease burden (1.2 million daily adjusted life years, DALYs; refs. [3,5]), linked to anaemia, malnutrition, and developmental impairment [3,6,7,8]. Despite the global impact of these parasites, research on their genomes has been uneven, leaving significant gaps in our understanding of some species, including *A. duodenale*—a species with unique biological, epidemiological characteristics, and clinical significance in humans.

Advances in genome sequencing have greatly enhanced our knowledge of some species of parasitic nematodes [9,10,11,12,13]. The genomes of *N. americanus* and *A. ceylanicum* were decoded approximately a decade ago [14,15], providing the first insights into hookworm biology, host adaptation, and drug targets. Although a fragmented draft genome assembly of *A. duodenale* has been publicly available in the National Center for Biotechnology Information (NCBI) database since 2015 (accession PRJNA72581) and was used in a previous publication [9], no high-quality genome has yet been reported for this species. The lack of a contiguous genome has significantly constrained comparative genomic analyses and impeded translational research opportunities. A recent review [12] highlighted the relative lack of progress in the area, noting also that some available hookworm genomes were derived from laboratory-maintained strains rather than wild-type, human-infecting populations. This aspect has raised concerns about genetic adaptations that might obscure biologically relevant traits and limit the development of new, effective interventions [12].

Addressing these gaps, the present study presents the first high-quality genome of *A. duodenale*, produced from single adult specimens archived in 70% ethanol since 1998. The successful extraction, sequencing, and assembly of DNA from long-preserved specimens not only fills a critical genomic gap but also establishes a precedent for harnessing archival material available in parasitology laboratories or museums. This achievement was made possible by the advent of third-generation sequencing technologies, which enable the sequencing of small amounts of DNA with high accuracy and long-read capabilities. Methods such as Oxford Nanopore and PacBio sequencing [16,17] have revolutionised genomic research by overcoming the limitations of short-read sequencing [18,19], facilitating the generation of contiguous genome assemblies even from degraded or low-input samples. Additionally, Hi-C sequencing [20,21] played a crucial role in the present study, allowing for the accurate scaffolding of genome fragments into chromosome-level assemblies, thereby achieving completeness and structural integrity of the *A. duodenale* genome.

By providing a robust genomic resource for *A. duodenale*, this work enabled comparative analyses with *N. americanus* and the related but free-living nematode *C. elegans*, improving our knowledge and understanding of specific differences, adaptations, host interactions, and potential therapeutic targets. The significance of this achievement extends beyond *A. duodenale*, demonstrating the feasibility of sequencing historical specimens and broadening the scope of genomic research to study previously unexplored parasites in well-curated archives. In doing so, this study contributes to an advance in hookworm genomics.

## 2. Results

### 2.1. Chromosome-Contiguous Genome for A. duodenale

**Figure 1:**
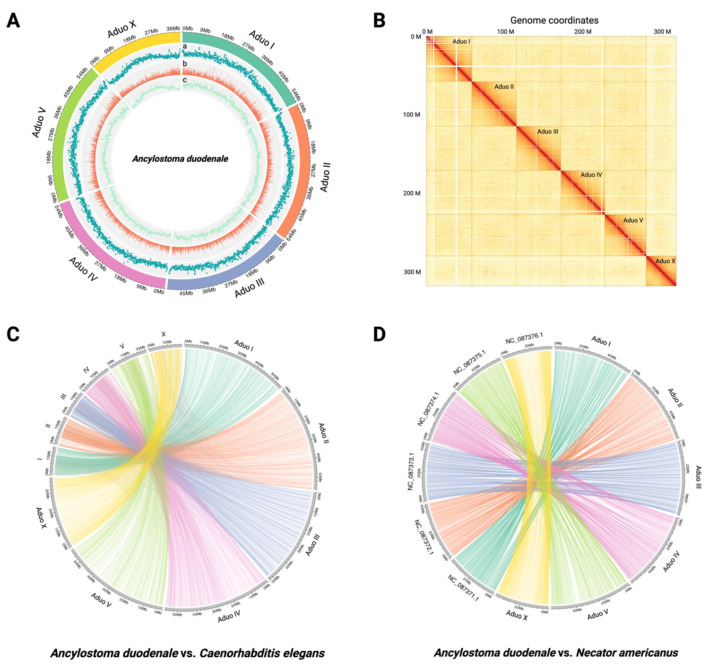
Genome assembly and chromosomal architecture of *Ancylostoma duodenale*, and comparative synteny with *Caenorhabditis elegans* and *Necator americanus*. (**A**) Circular representation of the six assembled *A. duodenale* chromosomes, showing distributions of (a) GC content, (b) gene density, and (c) repeat content using a 100 kb sliding window. Chromosomes are colour-coded, with autosomes (Aduo I to V) and sex chromosome (Aduo X) labelled. (**B**) Hi-C contact heatmap depicting chromosomal interactions and three-dimensional genome organisation. Strong intra-chromosomal contact domains are visible as dark red diagonal blocks, confirming the integrity of chromosome-scale scaffolding. (**C**) Syntenic relationships based on 7738 one-to-one orthologues between *A. duodenale* and *C. elegans*. Ribbon connections highlight conserved gene order and chromosomal rearrangements across the genomes. (**D**) Synteny between *A. duodenale* and *N. americanus* based on 9881 one-to-one orthologues, illustrating conservation and collinearity between the genomes of these two hookworm species.

In a first step, we obtained 10.23 Gb of PacBio sequence data with 32-fold coverage, 104.42 Gb of Oxford Nanopore data with 328-fold coverage, and 108.96 Gb of Hi-C data at 341-fold coverage from DNA from a single adult male worm (Table S1). From the long-read data obtained for the single male worm, we assembled a draft genome of 319 Mb (289 contigs; N50 = 1,772,226 bp; N90 = 547,225 bp; longest contig = 7,518,642 bp). By mapping Hi-C to this draft, we were able to assemble a chromosome-contiguous genome of 319 Mb consisting of five autosomal scaffolds and one sex-linked scaffold (Table 1; Figure 1A,B). A total of 3010 (96.1%) of 3131 complete benchmarking universal single-copy orthologues (BUSCOs) indicated a high-quality assembly for a nematode (Table 1); 75 (2.4%) were fragmented, and 46 (1.5%) were not detected, which is expected for a parasitic nematode (Table 1).

**Figure 2:**
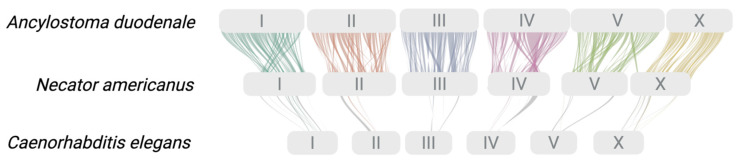
Syntenic relationships across the genomes of *Ancylostoma duodenale*, *Necator americanus*, and *Caenorhabditis elegans*. Syntenic blocks between the genomes of *A. duodenale* (top), *N. americanus* (middle), and *C. elegans* (bottom, strain N2) are shown, with chromosomes scaled by physical length. Each coloured ribbon connects homologous regions (syntenic gene blocks) between chromosomes, illustrating conserved macro-synteny across species. Conservation is evident between *A. duodenale* and *N. americanus* across all chromosomes (AduoI to Aduo V and Aduo X), reflecting their relatively close evolutionary relationship as human hookworms. More limited and fragmented synteny is observed between hookworms and *C. elegans*, consistent with greater evolutionary divergence. These results provide a framework for comparative functional analyses.

Individual chromosomes were designated in accordance with those of the free-living nematode *C. elegans* N2 strain (Figure 1C). A pairwise comparison of genomic sequences revealed a clear alignment of chromosomal regions among *A. duodenale*, *C. elegans*, and *N. americanus* (Figure 1C,D). Based on the pairwise comparison, GENESPACE analysis revealed marked orthology and synteny in shared alignment blocks (i.e., gene groups) between the genomes of *A. duodenale* and *N. americanus* but limited genuine synteny between either of these genomes and that of *C. elegans* using this approach (Figure 2).

### 2.2. The Annotated Genome

We inferred 20,015 protein-coding genes based on evidence of transcription in *A. duodenale* (Table 1). The average gene, CDS, and exon sizes are 5162, 1139, and 170 bp, respectively. Initiation and termination codons were defined, and the untranslated regions (UTRs) of 4416 messenger RNAs (mRNAs) were identified based on transcriptomic evidence. Of the 20,015 proteins predicted (Table S2), 14,884 (74.4%), 13,291 (66.4%), 11,272 (56.3%), and 12,388 (61.9%) protein sequences had most matches in the EggNOG, InterProScan, Gene Ontology (GO) and Kyoto Encyclopedia of Genes and Genomes (KEGG) databases, respectively (Table S2). Notably, 4535 (22.7%) proteins inferred had no homology to any known sequences in those databases. BUSCO assessment identified 2945 of 3131 (94.1%) complete orthologues and 84 (2.7%) as fragmented (Table 1); whether the 102 (3.3%) that were not detected are indeed absent from this parasitic nematode remains to be verified. This BUSCO score (94.1%) is similar to that obtained for the inferred proteomes of *N. americanus* (94.2%) (Table 1). A separate analysis revealed that 42.8% of the genome is repetitive, featuring prominent repeat elements, such as LTRs and LINEs of the retro-element category [22], and Tc1-IS630-Pogo of the DNA transposons category [23]. These repetitive elements were located predominantly in the central regions and at the ends of chromosomes (Figure 1A).

### 2.3. Genome Comparisons Between A. duodenale and N. americanus

The genome of *A. duodenale* (319.2 Mb; 20,015 protein-coding genes) is larger than that of *N. americanus* (234.5 Mb; 26,579 genes) (Table 1). Distinct co-linearity was observed between the respective chromosomes of these genomes (Figure 2); ~49.4% of the one-to-one single-copy orthologues (SCOs; *n* = 9881) were shared and syntenic between *A. duodenale* and *N. americanus* (Figure 1D). The mean gene lengths for *A. duodenale* and *N. americanus* were 5162 bp and 5570 bp, respectively, and the mean mRNA lengths were 5387 bp for *A. duodenale* and 5369 bp for *N. americanus*. The mean lengths of coding sequences (CDSs) were 1139 bp and 1083 bp for these respective species.

### 2.4. The Secretome of A. duodenale

**Figure 3:**
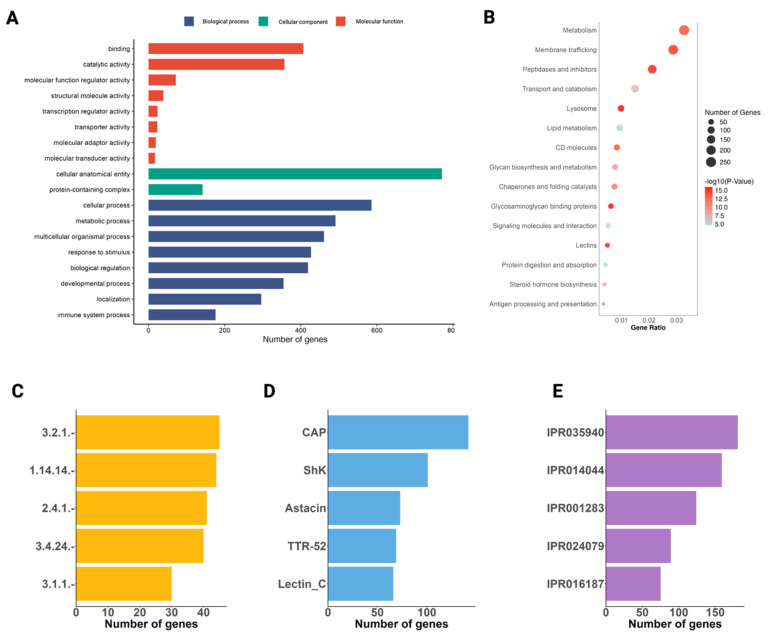
Functional classification of excretory/secretory (ES) proteins inferred from the *Ancylostoma duodenale* genome. Annotation of 3171 predicted ES proteins integrates Gene Ontology (GO), KEGG pathways, Enzyme Commission (EC) numbers, Pfam domains, and InterPro entries. (**A**) GO terms were assigned to 2374 proteins (19,189 terms), spanning biological process (BP; blue), cellular component (CC; green), and molecular function (MF; red). Top terms included cellular process and metabolic process (BP), binding and catalytic activity (MF), and cellular anatomical entity (CC), reflecting the extracellular nature of many ES proteins. (**B**) KEGG enrichment (1643 proteins, 666 KO terms) revealed significant involvement in pathways, including metabolism (257 proteins), membrane trafficking (226), peptidases and inhibitors (166), lysosome (77), glycan biosynthesis (60), and chaperone activity (58), among others. Bubble plots indicate gene ratio, gene count, and significance. (**C**) EC numbers were assigned to 400 proteins, dominated by hydrolases (3.2.1.–; *n* = 45), oxidoreductases (1.14.14.–; 44), glycosyltransferases (2.4.1.–; 41), metalloendopeptidases (3.4.24.–; 38), and ester hydrolases (3.1.1.–; 37). (**D**) Most frequent Pfam domains were CAP (*n* = 142), ShK (101), astacin (73), TTR-52 (69), and lectin_C (66), associated with host interaction and proteolysis. (**E**) InterPro signatures mirrored Pfam, with top entries, including IPR035940 (CAP), IPR014044 (ShKT), IPR001283 (astacin), IPR024079 (TTR-like fold), and IPR016187 (C-type lectin). Transcriptomic and proteomic evidence derived from Wong et al. [24].

Of the 3171 ES proteins inferred from the genome of *A. duodenale*, 2327 proteins were annotated using the integrated workflow (Table S4). Of these 2327 proteins, 1400 were assigned 19,189 GO terms (level 2) linked to 19 BPs, 3 CCs, and 19 MFs (Figure 3A; Table S4). For BP, cellular processes (GO:0009987; 36.7%) and metabolic processes (GO:0008152; 28.8%) were the largest sub-categories. Most proteins assigned to CC were linked to the cellular anatomical entity (GO:0110165; 39.1%), and those assigned to MFs were associated predominantly with binding (GO:0005488; 25.7%) and catalytic activity (GO:0003824; 16.4%) (Figure 3A).

In total, 1643 ES proteins were assigned to 666 KO terms, with enrichment observed in key biological pathways and processes. Significant enrichment was identified for metabolism (257 proteins, *p* = 3.81 × 10^−13^), membrane trafficking (226 proteins, *p* = 3.06 × 10^−14^), peptidases and inhibitors (166 proteins, *p* = 3.06 × 10^−14^), transport and catabolism (117 proteins, *p* = 1.36 × 10^−6^), lysosome (77 proteins, *p* = 4.60 × 10^−14^), lipid metabolism (73 proteins, *p* = 1.77 × 10^−4^), CD molecules (65 proteins, *p* = 4.83 × 10^−13^), glycan biosynthesis and metabolism (60 proteins, *p* = 9.31 × 10^−09^), chaperones and folding catalysts (58 proteins, *p* = 5.46 × 10^−11^), glycosaminoglycan binding proteins (48 proteins, *p* = 4.60 × 10^−14^), signalling molecules and interaction (40 proteins, *p* = 2.51 × 10^−4^), lectins (38 proteins, *p* = 3.06 × 10^−14^), protein digestion and absorption (33 proteins, *p* = 2.83 × 10^−4^), steroid hormone biosynthesis (30 proteins, *p* = 3.92 × 10^−7^), pattern recognition receptors (25 proteins, *p* = 3.06 × 10^−14^), apoptosis (24 proteins, *p* = 5.03 × 10^−4^), linoleic acid metabolism (24 proteins, *p* = 9.62 × 10^−10^), cytochrome P450 (23 proteins, *p* = 4.89 × 10^−10^), proteoglycans (19 proteins, *p* = 9.83 × 10^−4^), steroid biosynthesis (18 proteins, *p* = 6.45 × 10^−8^), and glycosaminoglycan degradation (17 proteins, *p* = 1.05 × 10^−8^) (Figure 3B). In addition, 400 proteins were assigned EC numbers relating to enzymatic activity; the major enzyme classes inferred were α-amino acyl peptide hydrolases (3.2.1.–; *n* = 45), oxidoreductases (1.14.14.–; 44), and hexosyltransferases (2.4.1.–; 41) (Figure 3C). The five most frequently identified Pfam domains in the dataset are CAP domain (*n* = 142), Shk (101), astacin (73), TTR-52 (69), and lectin_C (66). These domains are associated with a range of biological functions, including protein–protein interactions, enzymatic activity, and extracellular signalling. Their prevalence suggests a significant role in parasite biology, particularly processes such as host interactions, immune modulation, and/or tissue invasion.

### 2.5. Conserved Single-Copy Genes Associate with Critical Functions in Nerves, Gap Junction Channel or Pore Activities, Oxidative Phosphorylation, or Thermogenesis

Of the 9881 single-copy orthologues (SCOs) shared between *A. duodenale* and *N. americanus*, 96 are conserved genes (with an identity score > 0.99 at the protein level) (Table S3). Of these genes, 89 have orthologues (E-value: 10^−5^) in humans, and seven are uniquely present in *A. duodenale* but absent from humans (see Table 2). Of these seven genes, four encode presently uncharacterised proteins and two encode ES proteins (Aduo1G00000016964 and Aduo1G00000006435; Table 2).

The genes Aduo1G00000016964 and Aduo1G00000006435 encode ES proteins with distinct features. Aduo1G00000016964 contains a Ly-6-related domain, and its *C. elegans* homologue is involved in olfactory behaviour and response to odorant stimuli, with a subcellular localisation in the axon and neuronal cell body, suggesting a role in sensory signal transduction. Although Aduo1G00000006435 lacks a functional annotation, its *C. elegans* homologue is enriched in M cells, accessory cells, and intestine and muscle cells, with functional analyses indicating that its transcription is influenced by regulatory genes including *cyc-1*, *nuo-6*, and *qui-1* [25,26]. Furthermore, exposure compounds, including rotenone, stavudine, and zidovudine, have been shown to modulate the transcription of Aduo1G00000006435.

The genes Aduo1G00000008440, Aduo1G00000003031, and Aduo1G00000003651 also lack functional annotation. However, proteins encoded by *C. elegans* gene homologues (designated F21F3.6, *ssna-1*, and T27E4.7) are all inferred to be localised in the lysosomal membrane and are enriched in anatomical structures such as AVF, I2L, XXXL, anchor cells, and sensory neuronal cells [27,28,29]. These genes are under regulatory control by genes including *atfs-1*, *etr-1*, and *hsf-1*, and interactions with genes such as *nuo-6*, *cua-1*, and *atfs-1*. their expression is modulated by exposure compounds including allantoin, rifampin, rotenone, tunicamycin, and psoralens [27,28,29,30].

The genes Aduo1G00000011445 and Aduo1G00000012651 could be reliably annotated. On one hand, Aduo1G00000011445 encodes a structural component of gap junctions, containing an Innexin Pfam domain. KEGG pathway analysis (K22037) links this gene to signalling and cellular processes, particularly transporter systems (inx, zpg, ogre, shakB, and innexin). GO annotations revealed that Aduo1G00000011445 is associated with molecular transport, including transporter activity (GO:0005215), gap junction channel activity (GO:0005243), passive transmembrane transporter activity (GO:0022803), wide pore channel activity (GO:0022829), and gap junction hemi-channel activity (GO:0055077). Cellular component annotation indicates the localisation of the gene product to cell–cell junctions (GO:0005911), gap junctions (GO:0005921), and other cell junction structures (GO:0030054), and biological process annotations link it to transport (GO:0006810), ion transmembrane transport (GO:0034220), and cellular localisation (GO:0051179). The *C. elegans* homologue (designated R12H7.1) enables actin filament binding activity and gap junction channel activity, contributing to monatomic ion transmembrane transport and the regulation of locomotion. Consistent with the annotation of Aduo1G00000011445, R12H7.1 is primarily associated with gap junctions and transcribed in diverse tissue types, including anchor cells, muscle cells, neurons, phasmid sheath cells, and the somatic nervous system, indicating its functional relevance in intercellular communication and ion exchange. On the other hand, Aduo1G00000012651 encodes a profilin protein, which is inferred to be functionally associated with the KEGG pathway categories environmental information processing, membrane trafficking, and cytoskeletal proteins (K05759). GO annotation links this gene to actin binding (GO:0003779), supporting its role in cytoskeletal organisation and intracellular transport. The *C. elegans* homologue of this gene (designated *pfn-2*) associates with actin monomer binding activity, participating in muscle thin filament assembly, and localises to the cell cortex. Its transcription has been detected in the pharynx and spermatheca, indicating its potential involvement in reproductive and muscular functions (Table 2).

### 2.6. Structural Characterisation of ES Proteins Encoded by Aduo1G00000016964 and Aduo1G00000006435 and Their Association with Host Proteins

**Figure 4:**
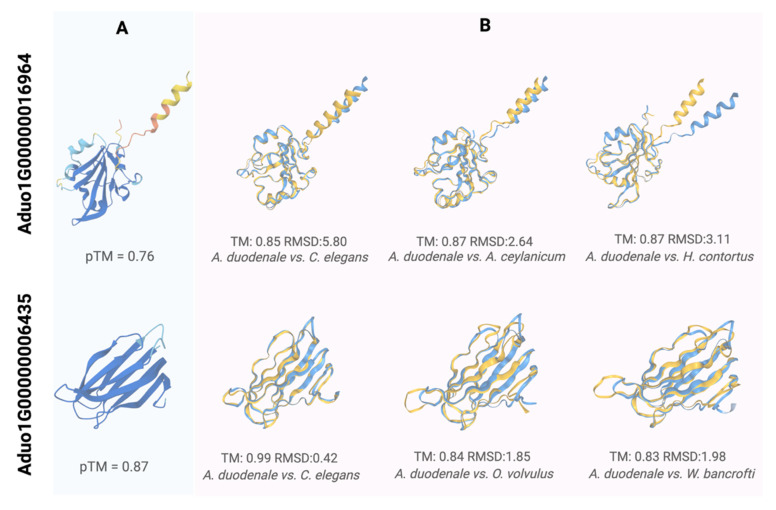
Structural characterisation and alignment of ES proteins encoded by single-copy genes Aduo1G00000016964 and Aduo1G00000006435 of *Ancylostoma duodenale*. This figure illustrates the predicted protein structures and their alignment with structural homologues in other species. (**A**) Predicted protein structures—The ribbon models show the AlphaFold3-predicted three-dimensional structures of Aduo1G00000016964 (top) and Aduo1G00000006435 (bottom). Confidence scores (pLDDT) are mapped to the colour gradient from red (low) to blue (high), with predicted template modelling scores (pTM) of 0.76 and 0.87, respectively, indicating good model reliability. (**B**) Structural alignment of *A. duodenale* protein Aduo1G00000016964 with its homologues in *C. elegans*, *A. ceylanicum*, and *H. contortus*, as well as Aduo1G00000006435 with its homologues in *C. elegans*, *O. volvulus*, and *W. bancrofti*. The Template Modelling score (TM) evaluates structural similarity, with values closer to 1 indicating a higher similarity. Root-Mean-Square Deviation (RMSD) represents the average atomic displacement between aligned structures.

**Figure 5:**
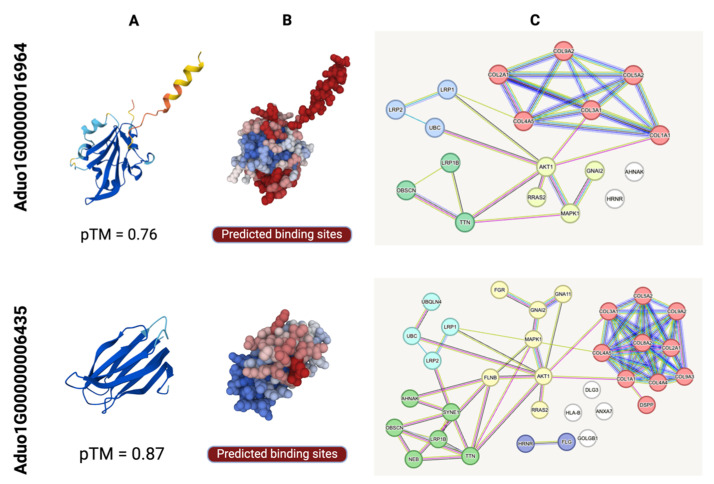
Structural characterisation and host–parasite interactome predictions for *Ancylostoma duodenale* proteins encoded by genes Aduo1G00000016964 and Aduo1G00000006435. This figure illustrates the predicted protein structures, surface binding sites and host–parasite protein–protein interaction (PPI) networks for two *A. duodenale* gene products with potential roles in host modulation. (**A**) Predicted protein structures—The ribbon models show the AlphaFold3-predicted three-dimensional structures of Aduo1G00000016964 (top) and Aduo1G00000006435 (bottom). Confidence scores (pLDDT) are mapped to the colour gradient from red (low) to blue (high), with predicted template modelling scores (pTM) of 0.76 and 0.87, respectively, indicating good model reliability. The structures reveal conserved β-strand-rich regions, indicative of immunoglobulin-like or β-sandwich folds. (**B**) Surface topology and binding site prediction—Electrostatic surface renderings highlight putative ligand or receptor interaction regions, with predicted binding pockets shown in red. These binding sites were inferred using computational methods (e.g., ScanNet), indicating molecular interfaces for host protein engagement. The central cavity in proteins encoded by Aduo1G00000016964 and the extended groove in that encoded by Aduo1G00000006435 indicate distinct potential interaction modalities. (**C**) Predicted interactomes against the human proteome—Protein–protein interaction networks show predicted human host interactors (All nodes) for each *A. duodenale* protein (Aduo1G00000016964 and Aduo1G00000006435). Interactions were inferred using graph neural network method (cf. [34]). Each edge represents a putative *Homo sapiens* protein. The light blue line represents known interactions from curated databases (cf. [35]), while the dark red line indicates known interactions derived from experiments. Predicted interactions related to gene neighbourhoods are shown in dark green, whereas those associated with gene fusion are represented by light red. The dark blue line signifies predicted interactions based on gene co-occurrence, and the light green line corresponds to interactions identified through text mining. Lastly, the black line represents predicted interactions related to co-expression. The interactome of these two proteins exhibits overlapping regions and can be categorised into four functional clusters (Table S5). The first cluster (red) is primarily associated with collagen chain trimerization, extracellular matrix structural integrity, and protein digestion and absorption, with key proteins including *COL1A1*, *COL2A1*, and *COL3A1*. The second cluster (yellow) is enriched in oestrogen-dependent nuclear events downstream of ESR-membrane signalling and labyrinthine layer blood vessel development, involving proteins such as *AKT1*, *MAPK1*, and *RRAS2*. The third cluster (green) is related to the M band and contains immune-related domains, with representative proteins including *TTN*, *LRP1B*, and *OBSCN*. The fourth cluster (blue) is associated with the positive regulation of lysosomal protein catabolism and the low-density lipoprotein receptor YWTD domain. Collectively, these results create a structural and system-level framework for understanding how *A. duodenale* might engage host biology through direct molecular mimicry or interference, providing hypotheses for future validation studies of host–parasite interactions.

We further characterised the ES proteins encoded by Aduo1G00000016964 and Aduo1G00000006435 in silico to explore host–parasite cross-talk [31,32]. The modelled structure of the first protein (Aduo1G00000016964; pTM = 0.76; Figure 4A) showed homology to ANCCEY_07682 (*Ancylostoma ceylanicum*; post-SET domain-containing protein; https://www.uniprot.org/uniprotkb/A0A0D6LT40/; ref. [15]; accessed 25 March 2025), HCON_00038200 (*Haemonchus contortus*; Quiver protein; https://www.uniprot.org/uniprotkb/A0A7I4Y1X8/; accessed 25 March 2025) and HOT-3 (*C. elegans*; Odr-2 homologue; https://www.uniprot.org/uniprotkb/Q8T3B0; ref. [33]; accessed 25 March 2025), suggesting roles in sensory signalling, enzymatic regulation, substrate binding, and/or catalysis. This protein was also predicted to interact with at least 18 human proteins, including (i) collagens (COL1A1, COL2A1, COL3A1, COL4A5, COL5A2, COL9A2) involved in extracellular matrix assembly; (ii) signalling proteins (AKT1, MAPK1, RRAS2) that regulate cell survival and proliferation; (iii) cytoskeletal components (AHNAK, OBSCN, TTN) important for cellular architecture; and (iv) proteins (LRP1, LRP1B, LRP2) associated with endocytosis, epithelial differentiation (GNAI2) or protein turnover (HRNR, UBC) (Figure 5; Table S5). Together, these findings suggest that Aduo1G00000016964 modulates host tissue structure and signalling to promote parasite invasion, infection, and/or survival.

The modelled structure of the second protein (Aduo1G00000006435; pTM = 0.87; Figure 4A) showed homology to ZK856.7 (*C. elegans*; https://www.uniprot.org/uniprotkb/Q23642/; ref. [36]; accessed on 25 March 2025). A0A2K6VYS3 (*Onchocerca volvulus*; novel immunogenic protein NIP-3; https://www.uniprot.org/uniprotkb/A0A2K6VYS3/; ref. [37]; accessed on 25 March 2025), and A0A1I8EZA8 (*Wuchereria bancrofti*; immunogenic protein 3; https://www.uniprot.org/uniprotkb/A0A1I8EZA8/entry accessed on 25 March 2025), supporting a potential immunogenic function. This protein was predicted to interact with at least 39 human proteins, including: (i) extracellular matrix components (COL1A1, COL2A1, COL3A1, COL4A4, COL4A5, COL5A2, COL8A2, COL9A2, COL9A3) involved in collagen synthesis and tissue integrity; (ii) cytoskeletal elements (TTN, OBSCN, FLNB, SYNE1, NEB); (iii) signalling proteins (AKT1, RRAS2, MAPK1, FGR, GNAI2, GNA11); (iv) proteins involved in protein turnover (UBC, UBQLN4, HRNR, FLG, DSPP, DLG3, ANXA7); and (v) HLA-B, associated with immune responses (Figure 5; Table S5). These findings suggest that Aduo1G00000006435 functions at the host–parasite interface, contributing to tissue remodelling, host signalling, and immunoregulation.

## 3. Discussion

We report the first chromosome-contiguous genome assembly for *A. duodenale*, a major human hookworm. This high-quality draft addresses a long-standing gap in hookworm genomics. Previously, resources for *A. duodenale* were limited to a fragmented draft (N50 of ~10 kb) covering ~63% of expected genes [12]. By leveraging modern long-read sequencing and scaffolding technologies, our assembly dramatically improves contiguity and completeness, yielding a robust reference genome for this species. This achievement exemplifies how new methodologies are enabling the field into a genomic “dawn” [12]. Notably, earlier hookworm genomes, such as those of *N. americanus* [14] and *A. ceylanicum* [15], relied on short-read data from laboratory-propagated worms, yielding thousands of scaffolds but incomplete gene sets. In contrast, the present *A. duodenale* assembly, derived from a single adult worm, achieves chromosome-scale scaffolding. This demonstrates the ‘power’ of applying low-input, long-read sequencing to parasitic nematodes and sets a new standard for hookworm reference genomes. The improved assembly will not only facilitate more accurate gene annotation and comparative analyses but also provide a foundation for exploring genome organisation (e.g., centromeres, telomeres, and synteny) that was previously intractable using fragmented assemblies.

The *A. duodenale* genome assembled here is ~330 Mb in size, placing it in the upper range for known hookworm genomes [12]. This is consistent with prior estimates for the species (~333 Mb; ref. [12]) and larger than the genomes of *A. ceylanicum* (~313 Mb; ref. [15]) and *N. americanus* (~244 Mb; ref. [14]). The expansion relative to *N. americanus* may reflect a higher repetitive content or species-specific gene family expansions. Indeed, repetitive elements constitute a substantial portion of the *A. duodenale* genome, similar to the ~23–30% repeat content reported for other hookworms [12,14]. Our assembly appears to have captured these repeats more completely, which likely contributed to the larger total size and emphasises the importance of long-read data for resolving complex genomic regions. We predict a total of ~20,000 to 21,000 protein-coding genes in *A. duodenale*, differing from the gene counts for *N. americanus* (~19,151 genes; ref. [14]) and *A. ceylanicum* (~27,000; ref. [15]). The majority of these genes of *A. duodenale* have identifiable orthologues in other hookworms and in the free-living model *Caenorhabditis elegans*, indicating that core nematode biological processes are conserved. However, consistent with observations for *N. americanus* [14], we found that *A. duodenale* has markedly reduced repertoires of certain gene families (e.g., G protein-coupled receptors and other sensory genes; Table S2) compared with the free-living nematode, *C. elegans*. Such contractions are typical of parasitic nematodes, reflecting adaptation to a host-dependent lifestyle, possibly with fewer environmental cues. Conversely, other gene families are expanded or uniquely present, as discussed in Section 4.3, highlighting the specialised biology of hookworms. Overall, the genomic architecture and content of *A. duodenale* are consistent with those of related hookworms and reveal species-specific features now accessible through a high-quality assembly.

Analysis of the *A. duodenale* gene catalogue reveals expansions in several protein families associated with parasitism, mirroring patterns observed for other hookworms [14,15]. In particular, genes coding for proteolytic enzymes were abundantly represented. We identified numerous proteases across multiple classes (metalloproteases, cysteine proteases, aspartic proteases), many of which are predicted to be excreted/secreted and lack close homologues in free-living nematodes (Table S4). Tang et al. [14] similarly indicated that 55% of proteases (325/592) genes in *N. americanus* encode secreted enzymes, including species-specific enzymes absent from *C. elegans*. Many of these proteases are thought to facilitate the hookworm’s blood-feeding habit and tissue migration by digesting host haemoglobin, serum proteins, and extracellular matrix components in the host. We also observed large families of protease inhibitors (e.g., cystatins, serpins, and Kunitz-type inhibitors) and antioxidant enzymes (e.g., peroxiredoxins), which can protect the parasite from host digestive enzymes and oxidative defences. This complements findings for the congener *A. ceylanicum*, in which transcription of protease inhibitors is upregulated during the establishment of infection [15]. Taken together, the enrichment of proteases and related factors in the *A. duodenale* genome accords with the parasite’s adaptation to a nutrient-rich (tissues and blood), yet hostile host environment. Other notable expansions include groups such as the SCP/TAPS (CAP domain) proteins, immune modulators, and anticoagulants, all of which play roles in host–parasite interactions. The presence and diversification of these parasite-specific gene families in *A. duodenale* are consistent with those reported in *N. americanus* and *A. ceylanicum* [14,15], reinforcing the notion that hookworms have evolved a unique molecular “arsenal” for a parasitic mode of existence.

Hookworms secrete a complex mixture of excretory/secretory (ES) proteins—the “secretome”—that is essential for their survival in the host. Our genomic analysis indicates that *A. duodenale* encodes a rich secretome, comprising more than 3000 proteins with predicted signal peptides or secretion motifs. These include the classical hookworm secreted protein families, such as the ASPs (*Ancylostoma* Secreted Proteins), cysteine-rich SCP/TAPS proteins, various proteases, lipases, and anti-coagulant factors. Many of these have been identified or characterised in other hookworm species and are known or inferred to be immunogenic or biologically important (e.g., [24,38,39]). For example, the ASP family was identified originally in *Ancylostoma* spp. and includes vaccine candidates, including *Na*-ASP-2 from *N. americanus* [14]. Although an early trial with recombinant *Na*-ASP-2 as a vaccine candidate was not successful [40], this protein family is likely central to hookworm biology as abundant secreted antigens. Consistent with *N. americanus*, which showed ~55% of its proteases to be secreted [14], *A. duodenale* also contains numerous secreted proteases that likely mediate blood digestion and immune evasion. Additionally, we identify secreted glycoproteins, mucins, and novel *A. duodenale*-specific ES proteins with unknown function, significantly expanding the catalogue of potential modulators released by the parasite. The secretome components identified here align well with prior comparative studies—for instance, Schwarz et al. [15] found that many of the genes uniquely upregulated by *A. ceylanicum* during infection encode excreted/secreted proteins (e.g., the “ASPR” subfamily of ASPs and various digestive enzymes). The relative conservation of these protein groups among hookworm species suggests they perform fundamental roles in host infection, such as facilitating skin penetration, mediating blood feeding, and modulating immune responses in the gut. Thus, the comprehensive genomic inventory of the secretome for *A. duodenale* not only confirms that this species deploys a similar suite of secreted effector molecules as its relatives but also provides a valuable resource for identifying conserved targets for interventional strategies. This has applied implications, as many secretome constituents are potential targets of host responses and have been proposed as vaccine or drug targets (e.g., [38,39,41]). By cataloguing the complement of these proteins in *A. duodenale*, we lay the groundwork for future functional studies of their roles in pathogenicity and host immunity.

An intriguing aspect of hookworm biology is the dynamic regulation of parasite genes in response to the host’s immune response or status. Although the present study focused on genome structure and content rather than transcriptomic experiments, insights from other hookworms can inform the discussion of *A. duodenale*’s gene regulation. Recently, Schwarz et al. [42] showed that *A. ceylanicum* can transcriptionally upregulate a large set of genes—including many ES proteins—when confronted with an intact host immune system. In controlled infection experiments in hamsters, more than 1900 hookworm genes were more highly transcribed in immunocompetent hosts compared to immunosuppressed hosts, specifically in the adult worm’s intestinal tissue [42]. Notably, this immune-responsive repertoire included 153 ES protein genes, encompassing both (apparently) rapidly evolving species-specific proteins and relatively conserved hookworm factors [42]. Such findings suggest that hookworms actively sense and respond to host immune attacks by boosting the production of, for example, anti-inflammatory proteins, protease inhibitors, and antioxidant enzymes in real time. It is likely that *A. duodenale* employs similar immunomodulatory tactics. Many of the secreted proteins encoded in the *A. duodenale* genome have homologues amongst the immuno-regulated genes of *A. ceylanicum* [42], indicating a relatively conserved strategy of immune evasion. For instance, if *A. duodenale* experiences stronger immune pressure in a particular host, one might expect an upregulation of its ASPs, cysteine proteases, and other key ES factors, analogous to the patterns observed in *A. ceylanicum*. This dynamic interplay highlights the importance of the hookworm secretome, not just as a static arsenal, but as a “responsive interface” with host immunity. In future work, integrating transcriptomic profiling of *A. duodenale* (for example, comparing worms from immunogenetically distinct hosts or different infection phases) would be invaluable in exploring these regulatory responses. Nonetheless, even from a genomic standpoint, the presence of extensive ES protein gene families in *A. duodenale* supports the proposal that this parasite is equipped to detect host defence signals and modulate its gene expression accordingly. Such plasticity likely contributes to the ability of hookworms to establish chronic infections, despite active host immune responses (cf. [42]). Understanding which *A. duodenale* genes are switched on in the face of host immunity could identify prime targets for intervention—because those genes are arguably the parasite’s most critical tools for survival in a hostile host environment.

Our work on *A. duodenale* comes at an important time for hookworm genomics [12]. After the initial publication of the *N. americanus* and *A. ceylanicum* genomes [14,15], relatively few new hookworm genomes have been added, leaving dozens of species with no genomic data [12]. The latter authors noted that as of 2024, only four genomes representing hookworm species were available—all derived from laboratory-maintained strains and sequenced and assembled using older technologies. This narrow sampling fails to capture the genetic diversity of wild hookworm populations and often results in draft assemblies of moderate quality [12]. The high-quality *A. duodenale* genome presented here helps to address both issues: it represents a parasite obtained from its natural host (humans), and it is a highly contiguous assembly, achieved through the application of contemporary sequencing methods. By sequencing and assembling the genome from a portion of a single worm (rather than a pool of many worms), we demonstrate that it is now feasible to generate reference-grade genomes even for species that cannot be readily obtained or maintained in the laboratory. This approach circumvents historical obstacles in hookworm research—for example, *A. duodenale* has long been difficult to propagate experimentally [12], with only limited success maintaining life cycles in puppies or other surrogate hosts (cf. [43]). Modern low-input protocols and portable (e.g., nanopore) sequencers may open the door to sequencing hookworms directly from field samples, including eggs or larvae from infected hosts [12]. A remaining technical challenge is achieving chromosome-level assemblies for numerous hookworms of animal and human health importance. In our case, the use of long-read sequencing, combined with scaffolding (e.g., Hi-C or linkage data), was crucial to infer chromosomes from contigs. The resulting *A. duodenale* assembly now enables synteny analyses and chromosomal comparisons that were previously impossible for members of the superfamily Ancylostomatoidea. Extending this approach to other hookworms should greatly enhance comparative genomics. However, some challenges persist, such as dealing with the high repeat content and heterozygosity often found among individuals of species and ensuring that reference genomes reflect the species’ natural genetic variability. Our work exemplifies how these challenges can be addressed using advanced technologies, setting a precedent for future hookworm and STH genome projects.

A high-quality genome for *A. duodenale* unlocks numerous opportunities for both fundamental research and translational applications. In terms of fundamental biology, researchers can now investigate the molecular underpinnings of traits that distinguish *A. duodenale* from other hookworms. For instance, *A. duodenale* is known to exhibit developmental arrest (hypobiosis) in human tissues and has a somewhat distinct epidemiological pattern compared with *N. americanus*. With a complete genome, researchers can now explore genetic factors or regulatory pathways that might contribute to these unique life history features. Similarly, the genome allows for more precise evolutionary comparisons among hookworm species, helping to clarify systematic relationships and host-range adaptations. Comparative genomics, using this assembly together with those of *N. americanus* and *A. ceylanicum*, might identify lineage-specific genes or divergent proteins that might relate to different host preferences or geographic distributions. From a practical standpoint, the *A. duodenale* genome could also significantly aid the identification of new drug and vaccine targets. Currently, hookworm infections are controlled mainly by anthelmintic drugs, but the reliance on mass drug administration has raised concerns regarding emerging drug resistance [14]. No approved vaccine is yet available for hookworm disease, despite several candidates entering clinical trials and recent success with a live vaccine for *N. americanus* (e.g., [44,45,46,47]). Genomic data provide a comprehensive list of the parasite’s proteins, many of which can be screened in silico for gene essentiality, a lack of homology to human proteins or surface/secreted expression—criteria that can help predict or identify promising therapeutic targets. Indeed, previous hookworm genomic studies have already highlighted potential target molecules: for example, an intestinal cysteine protease from *A. ceylanicum* was shown to confer partial protective immunity in an animal model [12], indicating the utility of gene catalogues in guiding vaccine development. In the *A. duodenale* genome, we have identified homologues of vaccine candidates proposed to date (e.g., ASPs, glutathione S-transferase, haemoglobinase enzymes like APR-1, and others), and their conservation or variation relative to *N. americanus* (the primary target of most vaccine efforts) can be assessed. This information could be useful for refining the selection of immunogens—for instance, ensuring that any vaccine for hookworms would be effective against both *N. americanus* and *A. duodenale*, which co-occur in many endemic regions. Additionally, the availability of a high-quality genome enables the exploration of drug resistance mechanisms; if benzimidazole or other drug resistance alleles have been reported in related nematodes, one can now locate and monitor such loci in *A. duodenale* individuals and populations. More broadly, having a genome available can accelerate the development of molecular tools (e.g., PCR diagnostics, CRISPR-based functional studies) for *A. duodenale*. In summary, this high-resolution genomic resource improves our ability to study the biology of *A. duodenale* in detail and supports the discovery pipeline for interventions—a critical step towards better control of hookworm infections and disease.

A chromosome-level genome for *A. duodenale* is an important milestone for hookworm research. It fills a gap in the genomic coverage of human hookworms and provides a solid foundation for future studies on parasite biology, evolution, and control. By integrating our findings with insights from previous hookworm genomes [14,15] and recent advances in transcriptomics [42], we have shown how *A. duodenale* compares with its relatives, and we propose how it interacts with its host at the molecular level. Importantly, this work exemplifies the transformative impact of new sequencing technologies on hookworm genomics. As Ilík et al. [12] poignantly described, hookworm genomics was at risk of stagnation, with few outdated reference genomes limiting the scope of comparative and applied research. The present study helps to usher in the “dawn” of a new era by delivering a high-quality reference for *A. duodenale*, demonstrating that even “challenging parasites” can be tackled with modern approaches. Looking ahead, we hope that the methods and findings presented here will catalyse further genomic projects on other hookworm species, including those infecting animals and those from field isolates, thereby broadening the genetic landscape available for analyses. Such expanded genomic resources will enable researchers to tackle profound questions regarding hookworm diversity, host-specific adaptations, and the genetic basis of virulence, pathogenicity, and drug resistance, for instance. Ultimately, the knowledge gained can be channelled into tangible benefits—from improved diagnostics to novel anthelmintics or vaccines—addressing a disease complex that continues to afflict hundreds of millions of people. This focus aligns with a *One Health* framework, the WHO NTD roadmap (2021–2030), and Sustainable Development Goal 3.3.

## 4. Materials and Methods

### 4.1. DNA Isolation

Adult males and females of *A. duodenale*, obtained from a human patient in Yiwu County, Zhejiang, China, were available from a previous study [48]. These specimens had been washed extensively in physiological saline, identified morphologically to species, and stored in ethanol at −20 °C for >27 years until use in the present study. Genomic DNA was isolated from single male or female worms using the Circulomics tissue kit (Baltimore, MD, USA), and the specific identity of each worm was confirmed molecularly by direct PCR-based (Sanger) sequencing of the second internal transcribed spacer of nuclear ribosomal DNA [48].

### 4.2. DNA Sequencing

Genomic DNA (6.25 ng) from a single male was subjected to whole genome amplification using the REPLI-g midi kit (Qiagen, Hilden, Germany), and the quantity and quality of this amplified DNA assessed in TapeStation system (Agilent 4200) using Genomic DNA ScreenTape (Thermo Fisher Scientific, Waltham, MA, USA). One aliquot of this DNA (20 μg) was used to construct a PacBio Revio/SMRT library, which was sequenced in the PacBio Revio Platform (at BGI, Hong Kong), with sequence data stored in the FASTQ format. A second aliquot of this DNA (1 μg) was used to construct an Oxford Nanopore SQK-LSK114 library, which was sequenced using a FLO-PRO114M flow cell in the Promethion 2 Solo Nanopore platform (Oxford Nanopore Technologies, Oxford, UK). The data were stored in the POD5 file format for subsequent “base-calling” and correction using the programme Dorado release v0.8.3 (Oxford Nanopore Technologies, Oxford, UK). Sequence reads were stored in the FASTQ format. In addition, two male and two female worms, whose specific identity were individually confirmed morphologically and molecularly, were pooled, weighed (3 mg), and then used for the construction of a library for subsequent Hi-C sequencing employing the Arima High coverage Hi-C kit (L/N2309050008; part no. A160162 v01; Arima Genomics, Carlsbad, CA, USA). This library was constructed using cross-linked and proximally ligated DNA, fragmented to 550–660 bp using an ultra-sonicator (M220; Covaris, Woburn, MA, USA), then assessed for quality using the TapeStation (as described above) and paired-end sequenced in one lane of a Nova Seq X (10B, 300 cycles; Illumina, San Diego, CA, USA), with short reads being stored in the FASTQ format. The quality of the sequenced reads was assessed using FastQC [49].

### 4.3. RNA Sequencing

Total RNA was isolated from midbody portions (5 mm) of single male and female worms using Tripure isolation reagent (Roche, Basel, Switzerland), and genomic DNA was digested using TurboDNase (ThermoFisher, Waltham, MA, USA). RNA quality was assessed using the Qubit RNA High Sensitivity Assay Kit (Life Technologies, Carlsbad, CA, USA), and the quality and integrity of this RNA were evaluated using the TapeStation employing RNA Screen Tapes (Agilent, Santa Clara, CA, USA). An aliquot (54 ng) was used to construct a cDNA library using the SQK-PCB114.24 kit and sequenced in a FLO-PRO114M flow cell in the same Promethion 2 platform (as used for DNA). Data were stored in the POD5 file format for subsequent “base-calling” using the programme Dorado release 0.8.3 (Oxford Nanopore Technologies, Oxford, UK) and sequence reads stored in the FASTQ format. The quality of the sequenced reads was evaluated using FastQC [49].

### 4.4. Genome Survey and Assembly

To obtain a draft genome for *A. duodenale*, PacBio and corrected Oxford Nanopore long reads from the single male worm were assembled using hifiasm v.0.19.8 (ref. [50]; https://github.com/chhylp123/hifiasm; accessed 20 December 2024). Then, haplotypic sequences were removed from the assembly using purge_haplotigs v.1.1.0 [51]. Chromosome-length scaffolds were obtained from the Hi-C dataset using chromap v.0.2.5 [52], yahs v.1.1 [53], HiContacts v.1.0 [54], and Juicebox v.2.20.00 [55] for scaffold curation. Gaps in scaffolds were closed using error-corrected long reads employing the programme DENTIST v.4.0.0 [56]. At each step, assembly results were assessed using QUAST v.5.2.0 [57] and BUSCO v.5.1.2 [58], and the parameters of the various software tools were optimised to achieve a chromosome-contiguous assembly.

### 4.5. Prediction and Functional Annotation of Protein-Coding Genes

First, custom repeat models, inferred from the *A. duodenale* draft genome using RepeatModeler v.2.0.4 [59], were masked in the assembled genome utilising RepeatMasker v.4.1.5 [60]. Then, gene models were predicted from the masked genome using a combination of bioinformatic tools or pipelines, including Braker v.3.0.3 [61,62], Funannotate v.1.8.1 [63], and PASA v.2.5.3 [64], supported by RNA and proteomic evidence in *C. elegans* (NCBI accession: GCA_000002985.3), *A. ceylanicum* (accession: GCA_000688135.1), and *N. americanus* (accession: GCF_031761385.1). To enhance annotations in Braker, we used high-quality RNA data and proteomes of the clade V nematodes *C. elegans*, *A. ceylanicum*, and *N. americanus*. The designation and orientation of individual chromosomes corresponded to those of *C. elegans inferred* using GENESPACE v.1.2.3 [65]. The completeness of the gene set was assessed using BUSCO v.5.1.2 for 3131 genes [58] and OMArk v.0.3.0 [66]. Genes/inferred proteins were annotated using an established pipeline [67], which incorporated InterProScan v.5.6.1 [68] and eggNOG-mapper v.2.1.9 [69], and presented in the GFF3 format and then refined to comply with NCBI submission requirements employing programmes AGAT v.1.2.0 (ref. [70]; https://github.com/NBISweden/AGAT; accessed 20 March 2025) and gffread v.0.12.7 [71].

### 4.6. Synteny and Genome Comparisons

Single-copy genes and one-to-one orthologues were identified in a pairwise manner among *A. duodenale*, *C. elegans* (version WS291), and *N. americanus* using OrthoFinder v.2.5.4 [72]. Subsequently, GENESPACE v.1.2.3 [65] was employed to assess synteny and orthology across the genomes of *A. duodenale*, *C. elegans* (version WS291), and *N. americanus*, focusing on alignment blocks within a length range of 30 to 50,000 kb. Circos plots were generated to visualise genomic relationships using shinyCircos v.2.0 [73] for optimal representation of the syntenic and orthologous regions across the three nematode species (clade V; ref. [74]).

### 4.7. Identification of Invariable Gene Sets, Inference of Essentiality, and Biological Pathway/Process Associations

All genes encoded in the *A. duodenale* genome were subjected to reciprocal homology analysis (protein level) against *N. americanus* using BLAST v.2.12.0 (ref. [75]; *p*-value: 10^−5^). Single-copy, one-to-one orthologues (SCOs) between *A. duodenale* and *N. americanus* were identified using OrthoFinder v.2.5.4 [72] (with default settings), and their amino acid identities were recorded. All SCOs were ranked from least to most variable based on their amino acid identities. Those with high identity (i.e., BLAST identity > 0.99; NEDDLE similarity > 90; ref. [76]) in amino acid sequences, for which no orthologue was detected in the human proteome (the natural host of *A. duodenale*; GRCh38.p14—accession: PRJNA31257; ref. [77]), were selected for further analyses. From this set of conserved SCOs, essential SCOs (eSCOs) were predicted/prioritised using an established machine learning (ML)-based approach [78,79], linked to *A. duodenale* chromosomes, enriched protein groups and biological pathways inferred using clusterProfiler v.4.0 [80] employing a custom script, and linking data to KEGG biological pathways, Pfam domains, and gene ontologies (GOs). The proteins inferred from selected eSCOs were then subjected to tertiary structure modelling using AlphaFold v.3.0 [81].

### 4.8. Identification and Annotation of Excretory/Secretory (ES) Proteins

We inferred and annotated the excretory/secretory (ES) proteins of *A. duodenale* using a recently established informatic workflow [67]. In brief, ES proteins were inferred from the nuclear genome of *A. duodenale*, with classical ES proteins predicted via Phobius v.1.0.0 [82,83] and SignalP 6.0 [84]. Proteins were required to contain a signal peptide but no transmembrane domain. Annotation was performed using eggNOG-mapper v.2.1.9 [69] and InterProScan v.5.57–90 [68]. Each tool provided complementary functional classifications, including gene ontology (GO) terms, enzyme commission (EC) numbers, and biological pathway associations. Gene ontology (GO) terms were retrieved from the GO-basic.obo file (release 19 September 2022), with pathway mapping performed via TB-tools v.1.0987663 [85]. Figures were produced using BioRender.com.

### 4.9. Structural Characterisation of ES Proteins Inferred for eSCOs, and In Silico Interactions with the Human Proteome Employing Machine Learning and Omic Data

We first identified eSCOs encoding ES proteins (see Section 4.7) with those predicted to be part of the secretome (see Section 4.8). The structures predicted from this protein were then used as queries for structural similarity searches in publicly accessible protein structure databases using Foldseek [86]. The databases accessed included: (i) BFVD [87]—a comprehensive repository of predicted viral protein structures; (ii) the AlphaFold Protein Structure Database (v4; ref. [88]); (iii) CATH50 (v.4.3.0; ref. [89])—a hierarchical classification of protein domain structures (50% identity, version 4.3.0) (iv) MGnify-ESM30 (v.1; ref. [90])—a large-scale structural dataset of metagenomic proteins; and (v) RCSB protein data bank [91]—a clustered version of the Protein Data Bank (PDB) containing high-resolution experimental structures. Inferred ES proteins with statistically significant (probability = 1) structural similarity to known proteins in one or more of these databases were used to annotate respective *A. duodenale* ES proteins encoded by eSCOs. Additionally, homologues in *Necator americanus* were identified in publicly available transcriptomic and proteomic datasets [24,39]. Subsequently, protein–protein interactions between these proteins and the human proteome were predicted using ProteinPrompt [34] to infer potential interactome relationships. Finally, *A. duodenale* proteins with a high probability (>0.9999) of interacting with human proteins were selected for interaction modelling employing STRING [35], and protein binding sites were predicted using ScanNet [92].

## Figures and Tables

**Table 1:** Genome assembly statistics for *Ancylostoma duodenale* and *Necator americanus*. Key assembly metrics include genome size, chromosome and scaffold counts, N50, GC content, and gap content (Ns). Gene prediction quality was assessed using BUSCO and OMark at the proteomic level. The *A. duodenale* assembly shows high contiguity and completeness, with fewer scaffolds and gaps than the *N. americanus* reference.

Genome Features	*Ancylostoma duodenale* (This Study)	*Necator americanus* (GCF_031761385.1) ^d^
Genome size (bp)	319,205,898	234,457,255
Number of chromosomes	6	6
Number of scaffolds	14	38
Largest chromosome (bp)	54,937,347	42,005,516
N50	56,497,455	38,919,484
GC content	42.95%	40.09%
Ns (gaps) ^a^	59,000	209,725
Number of gene models	20,015	26,579
BUSCO—genome (c; s; d; f; m) ^b^	96.1; 93.9; 2.2; 2.4; 1.5	96.7; 95.9; 0.8; 2.1; 1.1
BUSCO—proteome (c; s; d; f; m) ^b^	94.1; 91.1; 3.0; 2.7; 3.3	94.2; 93.1; 1.1; 1.2; 4.6
OMark—proteome (cs; ics; ct; uk) ^c^	69.6; 6.5; 0; 24.0	65.7; 4.7; 0; 29.6

^a^ Ns is the total number of uncalled bases in the assembly. ^b^ BUSCO results categorise genes into complete (c), complete and single copy (s), duplicated (d), fragmented (f) or missing (m) to assess the completeness and quality of a genome assembly; ^c^ OMark results categorise genes (at the proteome level) into “consistent” (cs), “inconsistent” (ics), “unknown” (uk) or “contaminant” (ct) to estimate the completeness of the gene-repertoire, estimate the proportion of accurate and erroneous gene models or detect possible contamination from other species. ^d^ Reference sequence available via the National Center for Biotechnology Information (https://www.ncbi.nlm.nih.gov/).

**Table 2:** Functional information for conserved single-copy genes (*n* = 7) in *Ancylostoma duodenale* that lack detectable homologues in the host (*Homo sapiens*). This table presents seven *A. duodenale* genes identified as relatively conserved, single-copy orthologues among *A. duodenale*, *Necator americanus*, and *Caenorhabditis elegans*, without detectable homologues in the human genome. For each gene, functional predictions are provided based on available annotations (e.g., Pfam, KEGG), secretome status, predicted protein structure, and relevant biological context from orthologues (e.g., in *C. elegans*) or computational analysis. These parasite-specific genes may represent novel targets for selective intervention.

*A. duodenale* Gene	*C. elegans* Orthologue	*N. americanus* Homologue (NCBI)	ES Protein	Transcriptomic Evidence in *A. duodenale*	ES Proteomic Evidence in *A. duodenale*	Functional Annotation (EggNOG; Pfam; KEGG)	Predicted Structure	Biological Context
Aduo1G00000008440	F21F3.6	XM_064179072	No	L3; adult (mixed sexes)	NI	NI	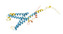	PL: Lysosomal membrane.
Aduo1G00000016964	*hot*-3	XM_013451594	Yes	L3; adult (mixed sexes)	NI	*Ly-6*-related	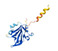	PF: Involved in olfactory behaviour and odorant response. PL: Axon and neuronal cell body.
Aduo1G00000003031	*ssna*-1	XM_064198606	No	L3; adult (mixed sexes)	NI	NI	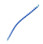	AC: Rotenone, rifampin, psoralens. AG: *atfs-1*, *etr-1*, *hsf-1*. EI: ABaraapapp, ABaraapppp, anterior hypodermis, germ line, neurons. PD: Phosphorylation site.
Aduo1G00000003651	T27E4.7	XM_013440797	No	L3; adult (mixed sexes)	NI	NI	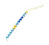	AC: Tunicamycin, psoralens, allantoin. AG: *nuo-6*, *cua-1*, *atfs-1*. EI: AVF, I2L, XXXL, anchor cell, sensory neurons.
Aduo1G00000006435	ZK856.7	XM_013445741	Yes	L3; adult (mixed sexes)	L3; adult (mixed sexes)	NI	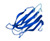	AC: Rotenone, stavudine, zidovudine. AG: *cyc-1*, *nuo-6*, *qui-1*. EI: M cell, accessory cell, intestine, muscle cell.
Aduo1G00000011445	R12H7.1	XM_013444081	No	L3; adult (mixed sexes)	NI	Gap junctions (component); innexin; K22037	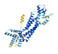	EI: Anchor cell, muscle cell, neurons, phasmid sheath cell, somatic nervous system. PF: Actin filament binding, gap junction channel activity; involved in monoatomic ion transmembrane transport, positive regulation of locomotion. PL: Gap junction.
Aduo1G00000012651	*pfn*-2	XM_013442503	No	L3; adult (mixed sexes)	NI	Profilin; K05759	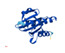	EI: Pharynx, spermatheca. PF: Actin monomer binding, muscle thin filament assembly. PL: Cell cortex.

Abbreviations: AG: Genes that influence or regulate the gene of interest (based on gene networks or RNAi data). AC: Compounds/chemicals that affect gene/protein expression or function. EggNOG: Evolutionary genealogy of genes: non-supervised orthologous groups. EI: Tissues/cell types where the gene is enriched or expressed (mainly based on *C. elegans*). ES protein: Predicted to be excreted/secreted (based on signal peptide or secretome analysis). KEGG: Kyoto Encyclopedia of Genes and Genomes (pathway and functional annotations). NI: No information available. PD: Protein domain (functional motif or modification site). PF: Predicted protein function (inferred from Pfam or orthologue function in *C. elegans*). Pfam: Protein family database (for conserved domains and motifs). PL: Predicted protein localisation (based on orthologues or structural models). Transcriptomic and proteomic evidence derived from Wong et al. [24].

## Data Availability

The complete genome assembly is available in the NCBI (National Center for Biotechnology Information) database under accession numbers PRJNA1246367 and SAMN47776112.

## Supplementary Materials

The supporting information can be downloaded at https://www.mdpi.com/article/10.3390/ijms26125576/s1.
